# Exploring Interactions in the Sickness Insurance System in Terms of Power and Trust

**DOI:** 10.1007/s10926-021-10017-4

**Published:** 2021-12-21

**Authors:** Elin A. Karlsson, Jan L. Sandqvist, Ida Seing, Christian Ståhl

**Affiliations:** 1 Department of Health, Medicine and Caring Sciences, Linköping University, Linköping, Sweden; 2 Department of Health, Medicine and Caring Sciences, Linköping University, Norrköping, Sweden; 3 Department of Behavioral Sciences and Learning, Linköping University, Linköping, Sweden

**Keywords:** Independent Medical Evaluation, Social Security, Work ethics, Work ability

## Abstract

*Purpose* Activation policies and efforts to reduce sick leave rates has influenced sickness insurance systems in Western countries, which has led to social security being more connected with work and attempts to expose malingering among the sickness absent. The aim of this study was to explore how power and trust are expressed by clients and stakeholders within the Swedish sickness insurance system. *Methods* This was a longitudinal qualitative study based on semi structured interviews and case files from 31 clients on sick leave in Sweden. Data was analyzed using a thematic analysis. *Results* The main theme ‘Acts of power and distrust’ illustrates how stakeholders’ express suspicions towards each other, and how clients need to demonstrate desire and efforts to return to work which other stakeholders verified. *Conclusions* The clients desire to prove themselves able to contribute to society was prominent in this study and power relations need to be acknowledged, in particular between client and the SIA. Further, to preserve citizens trust in the system, the system needs to demonstrate trust also in the clients.

## Introduction

A trend towards activation has been seen in many welfare systems, focusing on rapid integration for groups of people such as the unemployed, disabled and sick-listed, from inactivity and social exclusion towards working life [1–4]. Clients are expected to be “active” rather than “passive” recipients of financial benefits. Activation policies are political strategies aiming to reduce sick leave rates and the costs associated with it. Strategies used within activation policies have been either enabling or demanding, where the demanding ones often include restricting clients’ access to financial benefits [5].

Through policy reforms towards activation, the Swedish welfare state has become less universal and less generous [6], which is illustrated through restricted access to- and lower financial compensation. In a Swedish sickness insurance context, this activation approach has been expressed through a controlling and administrative turn by the Social Insurance Agency (SIA), by primarily assessing clients’ eligibility for sickness benefits rather than focusing on coordinating vocational rehabilitation. This is carried out by emphasizing the clients’ responsibilities towards society and by ensuring that social security is connected with work and activity [2].

Adverse associations of long-term sickness absence have been demonstrated in several studies in relation to increased mortality, future exclusion from work, low health, economic stress and dissatisfaction with the overall life situation [7–10]. Hence withdrawals of sickness benefits can be performed as a sign of caring, given the risks with sickness absence, but also as a way to reduce societal costs of sick leave rates and to fulfill political goals. In line with the broad shift towards activation and reducing the number of peopled on sick leave, studies show that case managers at the SIA function as sickness insurance guardians, preventing clients from remaining in the system [11]. It is important that the sickness insurance system is perceived as fair and legitimate, and that the SIA possesses the public’s trust [12]. The Swedish system is a comprehensive tax-funded social insurance system in which all citizens are insured in their ‘current state’ independent of what caused the work disability, as opposed countries that have adopted cause-based systems in which eligibility for sickness benefits require that the work disability be causally work-related [13, 14]. Citizens in comprehensive systems may have other expectations due to the wide coverage, hence trust is important and likely context dependent.

Studies of cause-based systems reported clients with accepted claims feeling that they were “treated like a criminal” [15] or feeling blamed for being an injured worker [16]. Studies in the Swedish comprehensive system reported clients feeling distrusted by the SIA and their own narratives being diminished [17]. Social insurance systems and its processes may have adverse effects on the clients’ health, as clients’ claims and prerequisites are often questioned [15]. This could be explained by stigma towards clients on sick leave [15, 18], imbalance of power between client and case manager and lack of social support to clients from other stakeholders [15]. The concept of moral hazard is often used to describe the effect of insurance incentives on behavior, for instance, the tendency for health insurance to increase the frequency of physician visits [18–21] or that having insurance could tempt people into immoral and potentially criminal behavior [19]. Moral hazard is often described in relation to malingering and feigned disease among workers [19], which has led to the legitimization of attempts by insurers and employers to expose these potential individuals [18]. Other studies demonstrate how regulation of the sickness insurance system is translated into moral practice as different stakeholders claim their right to direct the sick leave process and to justify their conclusions [22]. The values that the system is based upon, and the characteristics and prerequisites of the client may affect the client’s eligibility for sickness benefits [22]. These values are shaped in a context, dependent on the specific organization and are affected by policy and regulations [23] and thus, change over time. For instance, in the Swedish system the SIA in 2012–2015 focused on enhancing citizens trust in the authority, which changed in 2016–2018 as policies lead to a new focus on legal security. Hence, sickness insurance practice may vary, leading to a more or less generous system over time.

This area is important to explore further in relation to legal security, trust, the consequences it may have for clients’ wellbeing and for case managers’ discretion, and also in relation to the power imbalance between clients and other stakeholders. Power imbalance between clients and the SIA may put pressure upon the client to return to work before they perceive themselves as work ready, which may have adverse effects upon the client’s health and everyday life [24]. However, to our knowledge there is no consensus in research or practice that can pinpoint the proper time to initiate the process of returning to work or activity. However, in order for the authorities involved to preserve the trust of specific clients and citizens, this needs to be a transparent process characterized by individual adaptation and careful consideration rather than depending on time limits in the system, that instead may cause sickness presenteeism rather than sustainable return to work.

This study focuses specifically on how power and trust is expressed in the interactions between clients and welfare system actors. Power, in this study, refers to the ability to influence and affect others or resources [25], such as the sick leave process and other stakeholders. Trust refers to the belief in someone’s moral obligation to do something and trusting them to have the proper competence and intentions for it [26], such as clients relying on the SIAs responsibility to perform correct and just assessments.

## Purpose

The purpose of this study was to explore how power and trust are expressed by clients and stakeholders within the sickness insurance system.

## Materials and Methods

### The Swedish Sickness Insurance Context

Several stakeholders are involved in the Swedish sickness insurance system and have different roles. The SIA and its frontline staff have a key role in the implementation of sickness insurance policy. The case managers in SIA have the responsibility to assess clients’ eligibility for sickness benefits and to coordinate vocational rehabilitation. Assessments of client’s eligibility for sickness benefits exist in accordance with the so called ‘rehabilitation chain’ in which time limits for the durance of the sick leave determines on what criteria the assessment is carried out, with a few exceptions. For the first 90 days clients unable to do their regular work or any other temporary work that the employer can provide are eligible for sickness benefits [27]. From day 91 in the sick leave, clients are eligible for sickness benefits if they cannot do their regular work or any other work that the employer can provide. From day 181, clients are eligible if they cannot perform any work in the regular labor market [27].

In Sweden, case managers work in teams of eight to ten members who generally meet once a week to discuss difficult cases, sometimes also with the SIA’s own specialists (supporting quality in case managers assessments) and insurance physicians (physicians who do not meet clients but has an advisory function within the authority). Treating physicians within healthcare are responsible for submitting medical certificates that serve as the foundation for the case managers’ decision of granting or withdrawing sickness benefits. A sick note from the client’s treating physician is often sufficient in the early phase of the sick leave, but the client’s case manager can initiate a work ability evaluation when further information regarding the client’s work ability is required. These work ability evaluations play a central role in the continuation of the sick leave process and for the assessment of the client’s eligibility to receive sickness benefits. Work ability evaluations are performed by special units within health care on behalf of the SIA, as an objective one-time assessment, in which a physician, psychologist, occupational therapist and/or physiotherapist can be included.

### Study Procedures

This was a qualitative longitudinal study based on interviews and case files (the SIA’s documentation of the client's sick leave process) from clients on sick leave who were sent to a work ability evaluation on behalf of the SIA. Interviews and files were intended to complete each other and to provide the researchers with a richer picture of the clients’ sick leave process.

Six of the 35 evaluation units in Sweden performing work ability evaluations on behalf of the SIA chose to participate in this study. For those not participating, they stated reasons such as lack of time or that they have very few work ability evaluations and thus work primarily with other tasks. As part of a consecutive sample aiming at recruiting 30 clients, the units provided a total of 108 clients with an information letter. This was provided either by mail along with the invitation to the evaluation or provided to them personally by professionals during the evaluation. In the information letter to the clients, the study was described as focusing on the acceptability of assessments and decisions within the sickness insurance, including the experience of encounters and work ability evaluations and their perceived comprehensibility. Clients’ written consent and contact details were sent by mail to the first author who was then able to contact them by phone, or by text or e-mail if they did not answer the phone call. Informed consent was obtained both in writing and verbally, with information provided that included that participation was voluntary and that the researchers had no connection with the SIA and were hence unable to affect official decisions. This was considered important particularly due to the risk of clients feeling pressured if they believed that the researchers could affect their sick leave process.

Semi-structured telephone interviews were conducted in Swedish with 30 participants: first after one work ability evaluation and second after one official decision was communicated to the client by their case manager at the SIA. The first interviews were 30–100 min long, but mostly around 60 min, and examined the experiences of a work ability evaluation and the encounters with the involved stakeholders in the sick leave process. Examples of questions asked were to what extent they experienced that the evaluation was meaningful and contributed with information to the client’s case, and to what extent they considered the official decision upon sickness benefits to be reasonable, comprehensible and fair. The second round of interviews was conducted with 27 of the original 30 participants, one of whom was given the option to respond by e-mail due to difficulties managing another oral set of questions. Three participants did not respond to the invitation to the second interview, with no given reason. These interviews lasted for 10–40 min, mostly around 20 min, and examined their perceptions of the consequences after the evaluation and the contact with the involved stakeholders, in particular their case manager. The length of time between the first and second rounds of interviews varied between 4 weeks and 5.5 months. Two participants were interviewed a third time, for instance due to the case manager granting sickness benefits only temporarily while requesting further medical certificates. The interviews took place from November 2017 to June 2018, shortly after the clients had performed the work ability evaluation [1–14 days afterwards], and in most cases within the following week. One participant chose to share the file but not to participate in the interviews, hence this study consisted of 31 participants.

Client files were collected from the SIA during summer 2018 and included the complete documentation of the client’s sick leave case. The files are mainly documented by the case managers in the SIA, and hence mirror the case managers’ decision-making process and point of view, but also included the treating physicians’ medical certificates and the correspondence to and from the client and other stakeholders. In several cases, clients and the first author corresponded by text or e-mail between interviews in order to keep the first author updated about events in their sick leave process, and this correspondence was also included in the analysis. Thus, the data consisted of interview transcripts, texts, e-mails and SIA client files, representing primarily clients’ perspectives and partially other stakeholders’ perspectives.

### Participants

The participants were 12 men and 19 women, 36–64 years of age, representing several geographic parts of Sweden. The majority of the participants were on full-time sickness absence, but a few also worked part time while some had partial disability pension. Their disabilities represent a variety of diagnoses, often mental and musculoskeletal disorders and often combinations of several different diagnoses. Most of the participants were on long-term sickness absence (mean = 4.1 years) (see Table 1). Further, eight of the participants had fulfilled lower secondary school, while the distribution between those fulfilling upper secondary school (n = 11) and higher education (n = 12) was equal. There were no obvious gender differences in educational level and there was also an even distribution regarding blue/white collar professions among the participants. At the time of data collection, regulations for a one-year maximum of sickness absence had recently been removed (in 2016). This study’s many long-term sickness absent participants had been granted sickness benefits during this restrictive period, and thus had received a previously generous assessment.

**Table 1:** Characteristics of participants

No.	Sex	Birth year	Diagnostic category	SA/DP/work	Duration of current sick leave spell at the time of first interview, in years
1	F	1971	F, M, R	SA	3.5
2	F	1971	C	SA	6
3	M	1956	I, F	SA	4
4	M	1966	G, M	SA	6.5
5	F	1969	F, T, Q	SA	5.5
6	F	1977	F	SA & work	1
7	M	1954	T, H	SA	2.5
8	F	1965	L	SA	2
9	M	1980	F	SA	2
10	M	1970	M	SA	2
11	M	1970	F	SA	2.5
12	M	1982	R, M, T	SA	4
13	F	1965	F, R	SA	16.5
14	F	1959	M, F	SA & DP	0.3
15	F	1980	F	SA	2.5
16	F	1961	M, F	SA & DP	1
17	M	1976	M	SA	6
18	M	1962	M	SA	1.5
19	F	1981	F	SA	3
20	F	1965	M, G, F	SA	9
21	F	1980	F	SA	3
22	F	1956	M, F, T	SA	2
23	M	1956	M	SA	1.5
24	F	1967	I, M, J	SA	0.5
25	F	1963	M, F	SA & work	2
26	F	1956	F	SA	1.5
27	F	1957	I	SA & work	4
28	F	1970	M, T	SA & work	23
29	M	1984	F, L, K	SA	4
30	M	1960	G, M	SA	2
31	F	1954	F	SA & work	3.5

SA/DP/work refers to sickness absence (SA), disability pension (DP) and part-time work. Diagnostic categories refer to the diagnostic manual ICD-10: Mental and behavioural disorders (F), Diseases of the musculoskeletal system and connective tissue (M), Symptoms, signs and abnormal clinical and laboratory findings, not elsewhere classified (R), Neoplasms (C), Diseases of the circulatory system (I), Diseases of the nervous system (G), Injury, poisoning and certain other consequences of external causes (T), Congenital malformations, deformations and chromosomal abnormalities (Q), Diseases of the eye and adnexa/Diseases of the ear and mastoid process (H), Diseases of the skin and subcutaneous tissue (L), Diseases of the respiratory system (J), Diseases of the digestive system (K)

### Data Analysis

A thematic analysis was used, which is a qualitative analytic method that searches for themes or patterns within the data [28]. More specifically, a latent thematic analysis with an inductive approach was used in this study, in accordance with Braun and Clarke [28], in order to be able to explore underlying meanings of the data. All data was first read briefly by the first author, for one client at a time. Patterns of interest and ideas were noted and short summaries of each client’s case were written. The purpose of the summaries was to provide the researchers with an overview of each case. The amount of data was extensive, which facilitated putting codes into context during the analysis, and thereby increasing the researchers’ understanding of the clients’ descriptions by being able to relate to previous events. The complete data set was read through more carefully as data extracts were selected and coded into a table in one main document. In this step, some of the context of the data extracts was kept and included together with the data extract by stating, for instance: “from medical certificate (date)”, or “case managers’ documentation after telephone call with employer”. The next phase included sorting codes into potential themes [28], in which subthemes and main themes were identified. The themes were reviewed and refined by defining their names and content, while some were discarded, aiming for richer ‘story-book themes’ rather than summarizing ‘bucket themes’. In this phase, differences and similarities between themes regarding the participants gender, age, educational level and blue/white collar professions were briefly explored, although no clear patterns related to these characteristics were found in this study. The final step included writing the article and selecting quotations that were representative of each theme [28].

The first author performed these steps in consultation with the other authors, where continuous discussions lead to revisions such as new choices of quotes, condensation and reorganization of themes and text.

## Results

The analysis resulted in three subthemes: (1) *Highlighting the clients’ work ethics,*( 2) *Distrusting other stakeholders’ intentions and efforts within the sick leave process,* and (3) *Using instruments of power*, with one overarching main theme: *Acts of power and distrust*. The patterns identified include situations where clients and other stakeholders distrust each other. The clients feel that they have to prove their worthiness by stressing their desire to contribute to society, which other stakeholders tend to verify. Further, the involved stakeholders use different means to justify their conclusions. Instruments of power are central in the acts of power shown in this study and can consist of the interpretations of work ability evaluation results or the use of insurance physicians as means to state authority when deciding on the direction of the clients’ sick leave processes.

### Highlighting the Clients’ Work Ethics

Within this subtheme, clients stress their desire to and efforts towards work while other stakeholders, such as treating physicians, verify it. These stakeholders seem to feel the need to convey the image of the clients as morally trustworthy individuals who are not trying to misuse the system, and hence are not on sick leave by choice.

Descriptions of clients’ work ethics and a strong desire to return to work emerged, which was expressed in interviews through assertions of their passion and skills for their work, followed by grief and loss of identity when unable to.

*They (treating physicians at the hospital) said instantly, after looking at x-ray plates and after talking to me for a bit, that ‘Unfortunately we must tell you that you’ve had some severe strokes, you will never be able to work again, at least not full time’. And that was a shock to me because it was me, it was me and work. There (at work) I was really confident, sometimes somewhat confident in private contexts, but at work I was, there I knew what I was doing* [Interview with client 3].

In some cases, the strong desire to return to work illustrated the client’s expectations of work as a cure for illness. This was expressed for instance in e-mails to their case manager, stating that they want to initiate work training in order to get well faster, or in the interviews by stating that it is not good for anyone’s wellbeing to be home instead of working. Work as the solution to the client’s mental or physical difficulties causing work disability existed both among clients and, in some cases, treating physicians who expressed these opinions in medical certificates by stating that the client must be activated in order to get better. Other motivators for returning to work included perceptions of work as a strong social norm, and the stigmatization and shame attached to being sickness absent. Both in interviews and in documents from their files, clients highlighted that they did not choose to be work absent, that it was not their fault and that they obviously would work if they were able to. In the few cases where clients were not positive towards activation and returning to work, this seemed to be related to a lost faith in their ability, for instance due to too many failed attempts to return. This is illustrated in the files where clients are described as motivated during the first attempts to return to work or activity, followed by expressions of insecurity and frustration.

The desire to participate in and contribute to society was a prominent feature in this study, and some clients tried to find other ways to contribute when unable to work, which seemed to be closely linked to self-esteem.

*I help out at church sometimes, to the extent I can manage… And it means so bloody much when you are on sick leave… You feel that you are doing something good, that you, well that you mean something* [Interview with client 2].

The clients stated they did their very best during the work ability evaluation, often trying too hard and wearing themselves out, due to a desire to manage work and hoping to prove themselves able to contribute to society to some extent. The consequences of the efforts made by the clients in the work ability evaluation have been further described in another study [17], which was based on the same data as the present study. Clients who had their work disability questioned stated in the interviews that the case manager did not understand how hard they really tried during the evaluation and what physical and mental consequences they suffered afterwards. They stated that the case manager seemed to expect the clients’ work ability to be even better in real life than in the tests.

The clients’ treating physicians often verified their desire and efforts towards work, for instance by writing in the medical certificate that the client was highly motivated to work, had participated in all possible rehabilitation or had made several attempts to return to work without success. Other highlighted aspects from the files are the client’s own initiative in terms of treatment or work training and the emotions of failure when unable to work. In some cases, clients started work training that contradicted the physician’s recommendation due to their strong will to work. The case managers’ documentation had some similarities with the aspects noted by the treating physician, such as documenting clients’ own initiatives or emotions of being a burden to society. Further, employers highlighted the client’s emotions of failure or insecurity about not knowing beforehand if they would be able to work. Like treating physicians, employers tended to highlight the client’s strong desire to work and that the client had made several attempts. The efforts the clients made during evaluation tests are further verified at some evaluation units, where the assessment results could include statements from professionals observing how the client had overestimated their ability, pushed his or her limits during the tests or ‘crying and being sad when it does not work’.

### Distrusting Other Stakeholders’ Intentions and Efforts Within the Sick Leave Process

This subtheme describes explicitly expressed suspicion, i.e., distrust, towards other stakeholders’ agendas and competence, which commonly led to friction. Expressions of distrust are related to power relations, emerging through the stakeholders’ disagreement about who had the right to set the agenda, for instance whether the client should be on sick leave.

The most common form of distrust in this study, expressed in both interviews and files, was in regards to the frustration from clients and treating physicians when the treating physicians’ assessment was diminished by the SIA. This was due to physicians and clients feeling that the physician often know the client quite well while those who may never have met the client [the case manager and the insurance physician] had the most impact upon the client’s case and got to set the agenda. Distrust was also expressed by treating physicians towards the SIA by the physicians advising the client not to waste time and energy on protesting against the case managers’ decisions since they have no chance of winning. Some clients stated in interviews that this advice had been given to them also by evaluation units. Clients’ perceived powerlessness was described in terms of their own narrative being neglected and being questioned as malingerers when forced by illness or injury to stop working. This was primarily described in relation to the SIA, but in some cases also in relation to healthcare and the clients’ colleagues at work. The perception of having no say and not being trusted was described as insulting and was often expressed as negatively affecting the client’s mental wellbeing.

Clients described an exposed situation in relation to the SIA’s work ability evaluation, which they could not refuse to participate in without risking losing their sickness benefits. In interviews, they argued that they felt they could not refuse to do what the professionals required during the tests. Some clients expressed suspicion also towards the evaluation units, which were considered to have been ‘bought’ by the SIA and hence not being objective. Some had the perception that these evaluations were initiated when the case manager did not believe them and wanted them to be tested for cheating. However, some clients were positive in terms of them hoping for solid documentation proving the case manager wrong stating that they have nothing to hide, even though they believed the evaluation was a ‘cheat test’. Clients described their powerlessness in relation to the insurance physician and the case manager, often feeling forced to do more that they can manage such as initiating rehabilitation or work training.

Case managers’ medical competence was questioned primarily by treating physicians and clients, but sometimes also by evaluation units and other healthcare professionals. Case managers objected to such allegations in the files, where they stated they had the support they needed from insurance physicians and SIA specialists. In the interviews, clients described being affected media debates about the SIA, which enhanced their suspicions towards case managers agendas.

In one case, the medical competence of the insurance physician [and case manager] was questioned by the evaluation unit, which was rare:

*The only conclusion we [the evaluation unit] can draw regarding the somewhat unclear reasoning is that you do not understand the meaning of the chronic fatigue syndrome* [Response from evaluation unit when supplementary information was required by the insurance physician, client 21].

There were also cases where health care professionals questioned the interpretations made by the SIA although such objections were seldom listened to:

*It is not the role of healthcare, and they do not have the competence for this, to interpret a SIA work ability evaluation* [Statement by insurance physician, in the case of client 14].

### Using Instruments of Power

As a way of demonstrating one’s power or to justify one’s point of view as the correct one, different means were used by the stakeholders, which are considered here as instruments of power. Examples of such instruments were medical documents and alliances between stakeholders.

A commonly used instrument of power was the SIA’s work ability evaluation, where different stakeholders [primarily the SIA and healthcare] tended to refer to parts of the evaluation supporting their own opinions. Although healthcare professionals may mount an argument using evaluation results, this seldom affected case managers’ decision-making. Criticism of these evaluations was mainly expressed by clients [in interviews] or their treating professionals [in the documented files], questioning how an evaluation performed during a couple of hours could be given more importance than evaluations by other stakeholders, which were sometimes performed over the course of several months. It was clear from the files that case managers usually pursued results from the SIA’s work ability evaluation, tending to neglect other documents in the client’s case that may report other findings. There was one exception, where the SIA’s work ability evaluation demonstrated more significant difficulties compared to a work ability evaluation performed by healthcare. In this case, the case manager consulted the insurance physician who criticized the SIA evaluation and this discrepancy. This led to the case manager neglecting the results from the recent SIA evaluation by initiating vocational rehabilitation for the client in accordance with the evaluation from healthcare, i.e. pursuing results from the evaluation that supported a stricter assessment.

Another instrument of power was the treating physician’s medical certificate, which could be used by physicians both to comment on how the SIA’s actions had caused their client’s medical condition to worsen, and as a way for the physician to increase their status by highlighting their expertise within the area as evidence for their conclusions. Substandard medical certificates are used by case managers to justify the initiation of a work ability evaluation or the withdrawal of sickness benefits, which was perceived as incomprehensible by the clients who did not understand why they should be punished for their physicians’ poor writing skills. Other scenarios included when case managers delegated responsibility to clients to ensure that their treating physician submits a better medical certificate in order for the client to be able to receive sickness benefits.

*Then he (the case manager) said: ‘…I want your treating physician to submit a better medical certificate.’ ‘Yeah, well will you let him (the physician) know, then?’ ‘No, you do it,’ he said to me… and my physician was pissed off and said that this is between authorities, that my case manager at the SIA should contact him and not me since I should be kept out of this, and I agree with that. So… he didn’t want to write anything then but told me to tell X (the case manager) to call him instead. Sure, and then I am supposed to get in touch with him again and X (the case manager) says: ‘No that’s not it, but the physician is supposed to…’ I said: ‘Now you stop it… I can’t be the messenger between you two* [Interview with client 25].

The term ‘alliance’ refers here to when stakeholders have the same agenda or opinion and are working together to support each other to influence the sick leave process in a certain direction. Alliances were primarily seen between the client and their treating physician, characterized by the physician defending the client’s rights, but also in other constellations. In rare cases, treating physicians and case managers were allied, primarily when the physician recommended activation of the client. In one case, the case manager and the client had a clear alliance, as there was a conflict with the client’s employer. Insurance physicians and case managers was an example of an alliance within the SIA that it was difficult for other stakeholders to successfully object to. As noted in the files, work ability evaluations were often initiated in accordance with the insurance physician’s recommendations, and the evaluation results were usually consulted with them. They have a position of substantial power although they do not meet the clients, as highlighted by several clients in the interviews. Case managers often refered to regulations when justifying a withdrawal of sickness benefits. Although the clients may not know the content of the regulations, they expressed in interviews that official decisions are directed by the SIA management and the government, and hence not a particular case manager’s fault.

*Of course the SIA cannot articulate their decision so that those with granted or withdrawn sickness benefits can understand it, since there’s nothing comprehensible in it. Because it is not based on the principles of sickness insurance. It is based on top political demands* [Interview with client 5].

## Discussion

This qualitative study aimed to explore how power and trust are expressed by clients and stakeholders within the sickness insurance system. One important finding is that clients tried hard to prove themselves able to contribute to society, by wearing themselves out during work ability evaluations and expressing their motivation to work. This strong desire to work is demonstrated also in other studies [29–31], sometimes to the extent that when this does not work, the clients’ self-perception changes in a negative direction [30, 31]. The consequences of participating in a work ability evaluation is reported in terms of physical and mental exhaustion, and delayed consequences such as pain and fatigue leading to limitations in their everyday life and having to deal with emotions of failure and powerlessness [17]. In another study [32], clients highlighted their work ethics in their communication with their case manager by stating that they are doing their very best to recover and to contribute to society. In the present study, the clients state that case managers do not seem to understand how hard they really tried, assuming that they are ‘cheating’ and that their work ability is better in real life than in the tests. This could have fatal consequences if assessment results are interpreted as an underachievement and clients are assessed to be able to work if they are not.

There is research pointing to procyclical sickness absence, i.e., a generous sickness insurance system leading to higher sick leave rates and vice versa [33, 34]. However, the actual determinants of workers’ behavior (such as sickness absenteeism or presenteeism) and the length of their work disability are quite complex and include a variety of factors [18, 34]. Moral hazard assumptions have influenced sickness insurance systems and policy reforms in Sweden and other countries. In the Swedish system this is illustrated by activation and work being viewed as moral issues emphasizing increased individual responsibility for sick leave [35]. Assumptions of moral hazard were prominent in this study, for instance in terms of clients feeling distrusted and forced to prove their worthiness to receive sickness benefits due to work disability, while other stakeholders tended to verify and strengthen the image of the client as a genuinely disabled citizen doing their very best to recover. These experiences of distrust and suspicions in the system, may explain why clients and stakeholders needed to relate to this in diverse ways. While clients and some stakeholders verify the clients’ work ethics, case managers tend to become gatekeepers of the sickness insurance, leading to acts of power between stakeholders. This study may question some of the moral hazard assumptions within a sickness insurance context, since the results indicate that the work norm is strong also among the sick-listed. Findings in other studies confirm how clients’ work ethics and efforts to return to work are persistent [29, 36].

Case managers should possess character traits such as trust in the citizenry, but their professional ethics are as much about organizational design as they are about decision-making skills, since values are shaped in a context that is dependent on the specific organization [23]. Case managers are in a delicate situation as representatives for a system that may not always make sense to the citizens that it covers. Case managers need to follow regulations, work towards reaching political goals, and also explain and justify procedures and official decisions to clients in a comprehensible way. Their discretion is directed by regulations and policies, and they often need to rely on others’ expertise when decisions are made, such as insurance physicians and specialists at the SIA. Hollertz et al. [37] highlighted that team meetings between case managers and informal discussions between staff at the SIA created a normative practice where peer pressure and negotiation between team members encouraged case managers not to be too generous in granting sickness benefits. This teamwork was perceived by the SIA as an important part of achieving the organizational ideal of transparency, although the authority rather demonstrated internal transparency and external closure in relation to clients and other stakeholders. These findings are key components of the organizational mediation of activation policies [37], and can be related to another study where keeping an emotional distance from clients enabled case managers to enhance their organizational performance, keeping the self-image of oneself as a good, productive employee achieving organizational goals such as reducing sick leave rates by acting as sickness insurance guardians [11]. This study’s results illustrate client’s perception of the SIAs decision making process as a procedure characterized by incomprehensibility and distance. This applied to both case managers and insurance physicians, and also the evaluation units who are not included or responsible for the continuation of the client’s case as they serve as an objective assessor. Another study [17] highlights that a continuous dialogue between client and case manager facilitated the clients understanding and acceptability of procedures and the resulting official decision, as well as when sickness benefits were withdrawn. This is an important topic to explore further in order to achieve a more comprehensible sickness insurance that preserves its citizens’ trust.

It seems important for both case managers (as described in the studies above) and clients (as demonstrated in this study) to demonstrate proper work ethics, that they are doing a good job either as ‘good’ employees or ‘good’ absentees. Research on labor market policy and social assistance has shown similar findings, highlighting elements of activation and the importance of demonstrating a sufficient work ethic [38, 39]. This present study also demonstrates such findings in relation to the sickness insurance system. The sickness insurance system seems to play a controlling rather than supporting role by distrusting clients’ and other stakeholders’ statements, expressed in this study as acts of power between stakeholders. This is also demonstrated through the means used and the importance they are attributed, such as the power of SIA work ability evaluations and the value attributed to opinions of insurance physicians compared, to the treating physicians’ recommendations, which was observed also in another study [22]. Similar results regarding distrust and disadvantaged power positions between clients and other stakeholders have been demonstrated in sickness insurance systems also in other countries [40].

Distrust between stakeholders within the sickness insurance system may cause unnecessary insecurity and pressure for clients, hampering their opportunities to recover. This leads to the question whether a suspicious system may actually cause decreased work ability among clients on sick leave. Dembe and Boden [18] state that there are several examples of workers who decide to return to work before being work ready (i.e. sickness presenteeism) for fear of losing their jobs or due to economic insecurity, which may prolong the duration of their recovery as well as putting them at a greater risk of re-injury. A more generous and trusting system could instead help clients to stay home until they are more fully recovered [18] and increase the probability of them searching for a new suitable job due to the security of effective sickness insurance [41], i.e. a sustainable return to work beneficial to the client as well as to employers and society.

### Methodological Considerations

This study’s trustworthiness will be discussed using the terms ‘transferability’ and ‘credibility’ [42]. To facilitate clients’ participation in the interviews, they were given the opportunity to split the interviews into shorter sessions and the interviews were sometimes rescheduled several times depending on their expressed needs, which should be considered a strength in terms of transferability since this study includes clients with a variety of difficulties. Another strength is the rich data consisting of interview transcripts, client files from the SIA, and the correspondence between client and first author consisting of texts and e-mails in between interviews. However, the participants were long term sickness absent with a range of 1.5- 23 years of sick leave in their current sick leave which may have negatively affected this study’s’ trustworthiness and transferability. To investigate power and trust using a study population with extensive experience of the sickness insurance system may have affected this study’s results. For instance, several clients had already been extensively evaluated, which could potentially increase the experience of the SIAs recent work ability evaluation as unnecessary. An increase in the number of clients sent to work ability evaluations can in general be seen from day 181 in the sick leave, due to the assessment of eligibility being performed in relation to the regular labor market from that day; but these evaluations are used quite broadly by the SIA, as well as for these long cases. The long cases are often complex and include multimorbidity; but, in our opinion, power and trust are central regardless of the duration of sick leave. For instance, in contrast to shorter cases including temporary physical abilities, long term absentees may feel an increased need to highlight that they are not absent due to unwillingness to work, but because of long term illness and disability. The evaluation results become a powerful tool for the case manager to be able to make decisions in complex cases where obvious solutions are rare. Further, the clients in this study were given information in the information letter about this study’s focus on for instance the comprehensibility of processes in the sickness insurance. As in most research there is a risk that the participants who choose to participate are the ones most interested in the study’s topic, either those most critical or those who pleased, hence risking bias. There was no obvious overbalance of critical voices in this study, since most participants had both positive and negative aspects of their experiences to share.

The data represents the client’s perspective and their experiences to a large extent, while the case manager or physician perspective is not as prominent. Case managers have documented the client files and physicians’ perspectives are to some extent represented through medical certificates and other correspondence documented by case managers in the client files. But these stakeholders were not interviewed, which is a limitation in this study in terms of determining the magnitude of this study’s results. Further research could nuance these findings by also including case managers, treating physicians, insurance physicians and employers. In relation to other sickness insurance systems, such as cause-based systems, this study’s results can be useful and transferable regarding the importance of enhancing client’s comprehension of assessments and official decisions and also by acknowledging power relations between the client and other stakeholders. Since these matters were prominent in a comprehensive system such as the Swedish, they are also likely of great importance in cause-based systems where clients do not have the same coverage.

To strengthen the credibility of this study, several researchers were involved in the analysis by discussing ideas and themes. Further, the longitudinal design including follow up interviews allowed to build on previous conversations during the interviews which provided a richer picture of the clients’ experiences. The clients were recruited with assistance from the evaluation units, by mail along with the invitation to the evaluation or provided to the clients in person by professionals during the evaluation. In those cases where clients were provided the information letter in person, it may be considered a risky approach due to the power imbalance between client and assessor. It may have caused stress and discomfort with some clients if they felt pressured to participate, although such experiences were not apparent.

## Conclusions

This study demonstrates acts of power between stakeholders within the sickness insurance system, such as questioning other stakeholders’ competence, and how instruments of power were assigned diverse importance, depending on which stakeholder that used them. The opinions of insurance physicians and the results of SIA work ability evaluations was attributed most importance, and case managers’ interpretations of the evaluations took priority compared to interpretations made by healthcare professionals.

The study also questions assumptions of moral hazard, since clients state that they performed above their capacity during work ability evaluations in a desire to prove themselves able to contribute to society, rather than underachieving to secure their benefits. These efforts and the clients’ expressions of work ethics were further verified by other stakeholders, such as treating physicians and the professionals at the evaluation units.

The results from this study can be used to develop the sickness insurance system for instance by enhancing client’s comprehensibility for assessments and official decisions, by acknowledging power relations in particular between client and the SIA, and to preserve citizens trust in the system by demonstrating trust also in the clients.

## Data Availability

Not applicable.
